# Targeted protein degradation might present a novel therapeutic approach in the fight against African trypanosomiasis

**DOI:** 10.1016/j.ejps.2023.106451

**Published:** 2023-04-22

**Authors:** Ammar Usman Danazumi, Ibtida Tabassum Ishmam, Salisu Idris, Matylda Anna Izert, Emmanuel Oluwadare Balogun, Maria Wiktoria Górna

**Affiliations:** a Biological and Chemical Research Centre, Department of Chemistry, University of Warsaw, Warsaw, Poland; b Faculty of Chemistry, Warsaw University of Technology, Warsaw, Poland; c Department of Biochemistry, Ahmadu Bello University, Zaria, Nigeria; d African Centre of Excellence for Neglected Tropical Diseases and Forensic Biotechnology, Ahmadu Bello University, Zaria, Nigeria

**Keywords:** African Trypanosomiasis, Proteasome, Ubiquitination, Targeted Protein Degradation, PROTAC, TrypPROTAC

## Abstract

African trypanosomiasis (AT) is a hemoparasitic disease caused by infection with African trypanosomes and it is prevalent in many sub-Saharan African countries, affecting both humans and domestic animals. The disease is transmitted mostly by haematophagous insects of the genus *Glossina* while taking blood meal, in the process spreading the parasites from an infected animal to an uninfected animal. The disease is fatal if untreated, and the available drugs are generally ineffective and resulting in toxicities. Therefore, it is still pertinent to explore novel methods and targets for drug discovery. Proteolysis-targeting chimeras (PROTACs) present a new strategy for development of therapeutic molecules that mimic cellular proteasomal-mediated protein degradation to target proteins involved in different disease types. PROTACs have been used to degrade proteins involved in various cancers, neurodegenerative diseases, and immune disorders with remarkable success. Here, we highlight the problems associated with the current treatments for AT, discuss the concept of PROTACs and associated targeted protein degradation (TPD) approaches, and provide some insights on the future potential for the use of these emerging technologies (PROTACs and TPD) for the development of new generation of anti-*Trypanosoma* drugs and the first “TrypPROTACs”.

## 1. Introduction

African trypanosomiasis is a kinetoplastid parasite disease that affects both humans and domestic animals. While *T. brucei gambiense* and *T. brucei rhodesiense* are responsible for the Human African trypanosomiasis (HAT), *T. brucei, T. vivax,* and *T. congolense* account for the majority of cattle infections (Nagana). Trypanosomiasis is transmitted by the tsetse fly of *Glossina spp* and is endemic in at least 36 African countries (World Health Organization, 2020). If left untreated, the disease usually results in death, and major symptoms include anemia and severe weight loss (Balogun et al., 2014). It is estimated that about 70 million people are at risk of contracting HAT worldwide, and about $4.7 billion is lost per year due to animal infections (Mulenga et al., 2020; Simarro et al., 2012). Although the reported annual HAT cases have drastically reduced to less than 1000 cases (World Health Organization, 2020), this may not reflect the actual situation as cases are under reported due to poor diagnosis of the disease, especially for the affected population, and as such, results in under-testing. The situation becomes even more worrisome considering the zoonotic potential of the disease and emerging resistance to the available trypanocides (Fairlamb and Horn, 2018; Umeakuana et al., 2019). Although host innate immuno-logical response develop against the parasites during AT, the parasite quickly switches the expression of its surface antigen, giving rise to a new population of resistant parasites expressing distinct variable surface glycoprotein (VSG) (Cross et al., 2014). This has restricted the development of a vaccine against trypanosomes, and therefore chemotherapy remains the best option for managing AT. The available trypanocides used for treatment are either ineffective at a particular stage of the disease, toxic, or expensive for the affected population, and drug resistance to these trypanocides is fast spreading and recorded in about 21 African countries (Giordani et al., 2016). Although efforts towards vaccine design and drug discovery against AT have been intensified and some good drug and vaccine candidates (such as the invariant flagellum antigen from *T. vivax*) are reported (Autheman et al., 2021; Balogun et al., 2019), there is a need to explore alternative therapeutic strategies in order to combat this disease.

PROTACs present a new class of a therapeutic strategy possessing several advantages over classical small molecule inhibitors. They elicit their action by inducing proteasomal degradation of the protein of interest (POI). The PROTAC technology has been most successfully exploited in the treatment of cancer, but its newest frontier and promise may lay in combating infectious diseases (Békés et al., 2022), and in particular, its potential in the treatment of parasitic diseases has yet remained largely unexplored. Herein, we briefly discuss the problems associated with vaccination and chemotherapy against AT, introduce the concept of PROTACs and other targeted protein degradation (TPD) approaches, and suggest possible ways in which these innovative molecules can be considered for the treatment of AT.

## 2. Vaccine design against African trypanosomiasis

Vaccine design is considered the best approach for eradicating infectious diseases, and indeed the worldwide elimination of smallpox has been achieved through vaccination. Several vaccination approaches have been considered in trypanosomes, but vaccination against these parasites remains an ambitious goal. One of the earlier vaccine targets is the variable surface glycoprotein but targeting this molecule proves to be almost impossible due to antigenic variation (Black and Mansfield, 2016). Owing to the importance of the flagellar pocket, which is an invagination of the membrane involved in essential cellular processes, including antigenic variation, trypanosomal proteins embedded within this feature become suitable vaccine targets. Indeed, 60% protection from *T. congolense* infection was observed in cattle immunized with an antigen from the flagellar pocket of *T. brucei rhodesiense*. However, the protection was short-lived and non-effective when the animals were challenged with a high dose of the parasite (Mkunza et al., 1995). A similar observation was recorded when mice were immunized with a DNA plasmid encoding an invariant surface glycoprotein (ISG). Animals immunized with DNA vaccine carrying the ISG displayed 40% protection which also appeared to be temporary and only at low dose challenge (Lança et al., 2011). Several other vaccine candidates were identified, including cytoskeleton components (actin and tubulin), membrane-associated sialidases, cation ATPases, and a cysteine protease (congopain). However, most of these candidates provide partial or no protection even at low parasitemia challenges (Greca and Magez, 2011). Though there is speculation that the failure of these candidates may be attributed to the mouse model and might be an over-exaggeration of what would be observed in humans, trypanosomes indeed possess multiple strategies to escape destruction by their host, so that they were referred to as “the ultimate immune destroyers and escape artists” (Greca and Magez, 2011). Without any assistance, the host recruits both cellular and humoral immune responses during the infection, but the parasite undermines these efforts through several mechanisms. Starting with differentiation from the infective metacyclic form (MCF) to the bloodstream forms (BSF), the parasite randomly expresses VSG genes in each cell, resulting in a population of trypanosomes with overwhelmingly different surface proteins that ensure parasite survival (Graham and Barry, 1995). Trypanosomes also display very rapid and efficient endocytosis that allows the clearance of antibody-bound VSG, thereby preventing antibody-mediated destruction (Field and Carrington, 2009). Through the secretion of selected enzymes and metabolites like adenylate cyclase and indole-pyruvate, *T. brucei* avoided the trypanolytic effect of tumor necrosis factor (TNF) and nitrogen oxide (NO) generated due to the activation of macrophages (Drennan et al., 2005). These, together with the impairment of host B-cell memory by trypanosomes (Radwanska et al., 2008), rendered most of the vaccine design efforts unsuccessful. Recently, however, our group reported a putative multiepitope vaccine candidate against *T. brucei gambiense* designed from a collection of the parasite’s trans-membrane proteins (Danazumi et al., 2022). Additionally, a vaccine candidate from the invariant flagellum antigen of *T. vivax* has been proven to induce protective immunity in a mouse model of AT (Autheman et al., 2021). These have restored hope in achieving vaccinations against trypanosome infections.

## 3. Chemotherapy against African trypanosomiasis

While some drugs are available for HAT treatment, none of them is free from multiple limitations. For example, suramin and pentamidine are suitable medications for the treatment of *T. brucei rhodesiense* and *T. b. gambiense* infections. However, they are only effective at the first stage of the disease and are associated with high toxicity (Field et al., 2017). Melarsoprol is a drug used to treat the second stage of HAT and is potent against both human parasites but suffers from high toxicity issues as an arsenic derivative. Drug-related mortality is recorded in roughly 5% of patients treated with melarsoprol, and resistance to this drug has widely spread since the 1990s (Fairlamb and Horn, 2018). Eflornithine is another effective drug against the second stage of HAT. Yet, eflornithine usage is limited by its high cost, by a complicated use, longer duration of administration of up to 56 intravenous infusions, and by the fact that it is only active against *T. b. gambiense* (Burri and Brun, 2003). The introduction of an anti-Chagas drug, nifurtimox as a combination therapy with eflornithine, known as nifurtimox-eflornithine combination therapy (NECT) makes the treatment for the second stage of HAT less expensive and reduces the duration of the administration compared with the use of eflornithine alone (Priotto et al., 2009). Notwithstanding, NECT shares the same weaknesses as eflornithine treatment and has been the only progress made in the past 28 years of HAT research (Cullen and Mocerino, 2017). However, Pafuramidine, a diamidine used to treat pneumonia, has been repurposed to treat HAT and has exhibited equal potency with less toxicity than pentamidine (Burri et al., 2016). Pafuramidine has reached phase 3 clinical trial and may restore hope in searching for potent and less toxic trypanocides against HAT. The main trypanocides used for the treatment of animal trypanosomiasis (AAT) are diminazene aceturate and isometamidium. While these drugs are considered outdated and inadequate due to serious side effects, drug resistance to them is fast spreading and recorded in about 21 African countries (Giordani et al., 2016). It is evident that both HAT and AAT lack safe, effective, and easily accessible drugs. The emergence of resistance coupled with the zoonotic nature of these parasites necessitates the continuous investigation of alternative treatment approaches.

## 4. The ubiquitin-proteasome system in African trypanosomes

**Fig. 1.**
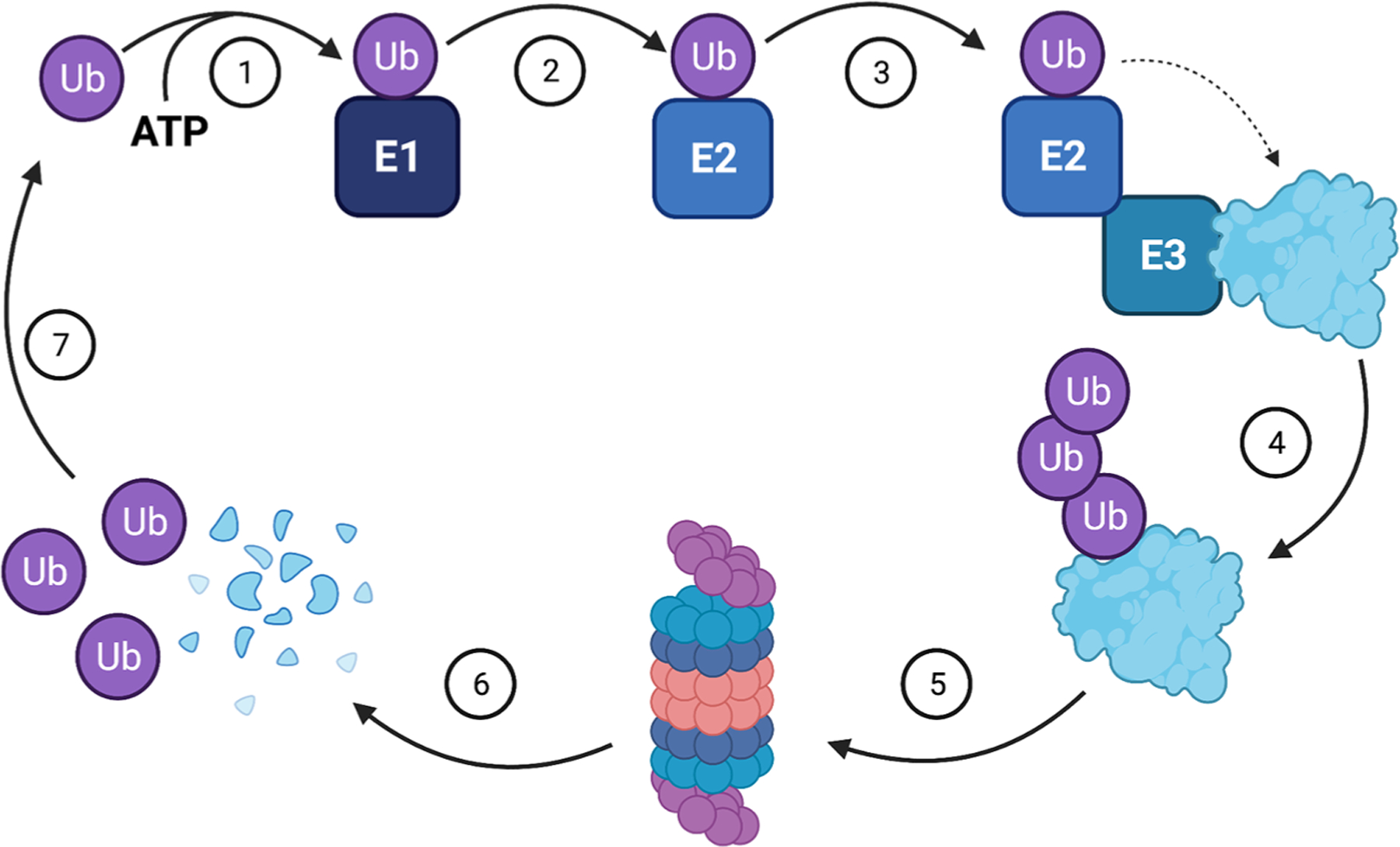
Mechanism of ubiquitin-mediated proteasomal degradation of cellular proteins. **(1)** Ubiquitin activation by ubiquitin-activating (E1) enzyme. **(2)** Ubiquitin transfer from E1 to a ubiquitin-conjugating (E2) enzyme. **(3)** Formation of ubiquitin-E2-E3-substrate complex. **(4)** Ubiquitination of the substrate. **(5)** Recognition of ubiquitinated substrate by the 26S proteasome. **(6)** Degradation of the substrate by the proteasome. **(7)** Ubiquitin is reutilized for the next cycle of substrate activation. *Figure Created with* BioRender.com.

The ubiquitin-proteasome system (UPS) is a homeostatic protein degradation pathway in eukaryotes that regulates the half-life of proteins through reversible posttranslational modification. It utilizes a 76-amino-acid polypeptide, ubiquitin, and begins its function when the E1 ubiquitin-activating enzyme is charged with a molecule of ubiquitin which is subsequently transferred to the E2 ubiquitin-conjugating enzyme (Fig. 1) (Hershko et al., 1983;). Ubiquitin is finally attached to the target protein by E2 through the assistance of E3 ubiquitin ligase. E3 is mainly responsible for recognizing and recruiting the target protein for ubiquitination; however, it also accepts ubiquitin from E2 and directly catalyzes its conjugation to the protein of interest. The 26S proteasome then takes up the polyubiquitinated substrate for proteolytic degradation (Kerscher et al., 2006).

UPS is generally an indispensable pathway in eukaryotes. It is involved in regulating many cellular processes, including cell cycle progression, repair of DNA, immune response, gene transcription, and signal transduction (Tanaka, 2009). This system is ubiquitously found among eukaryotes, and trypanosomes are no exception. The 26S proteasome was confirmed to be present in trypanosomes (de Diego et al., 2001), albeit at first as a 20S proteasome core subunit (González et al., 1996; Shao-Bing et al., 1996). It was reported to be crucial for cell growth and degradation of ubiquitinated proteins in these parasites (Li et al., 2002). In *T. cruzi,* for example, the differentiation of trypomastigotes to amastigotes was inhibited by a proteasome inhibitor lactacystin, (González et al., 1996). This demonstrated the importance of the proteasome pathway during cellular differentiation in *T. cruzi*.

While polyubiquitin genes and ubiquitinated proteins have long been identified in *T. brucei* (Lowrie et al., 1993; Wong and Campbell, 1989), it was not until recently that evidence began to accumulate regarding the mechanism and substrate proteins degraded through the ubiquitin-proteasome system in the parasites. A comparative genomic study recently identified 269 putative ubiquitin-proteasome pathway proteins (ubiquitin and ubiquitin-like proteins, E1, E2, E3, deubiquitinases) in *T. cruzi* and the conservation of many of their homologs across several species of trypanosomes, including the causative agents of both sleeping sickness and Chagas disease (Gupta et al., 2018). The putative trypanosome UPS proteins were also predicted to be widely localized across different organelles of the cell, such as the nucleus, cytoplasm, outer mitochondrial membrane, peroxisome, cytoskeleton, and plasma membrane, suggesting involvement in the degradation of a broad range of targets (Gupta et al., 2018). It was also demonstrated that *T. b. brucei* ISG65 and ISG75 were turned over through ubiquitination of their respective cytoplasmic domain lysine residues (Chung et al., 2008; Leung et al., 2011). Two *T. brucei* orthologues of human deubiquitinases Usp7 and Vdu1 designated TbUsp7 and TbVdu1 were confirmed to be expressed in the parasite, and their knockdown increased the turnover of ISG75 and ISG65 (Zoltner et al., 2015). In addition, the receptor of glycosomal matrix proteins, TbPEX5, is regulated by monoubiquitination and polyubiquitination in eukaryotes like yeast and human, of which the latter happens only when the protein is destined for proteasomal degradation. Monoubiquitinated TbPEX5 was similarly detected in *T. brucei* and interacted with TbPEX4 ubiquitin-conjugating enzyme (Gualdrón-López et al., 2013). Many of UPS features known from studies on yeast or human cells appear to be conserved in trypanosomes, including proteasomal involvement in the mitochondrial protein quality control in *T. brucei* (Dewar et al., 2022), a stress response pathway which in the case of trypanosome is triggered by the release of ubiquitin-like protein TbUBL1 from the nucleus.

On average, around 1.39% of the trypanosomal proteome was predicted to be involved in UPS (Gupta et al., 2018), which percent-wise is almost on par with the *Homo sapiens* genome which among others encodes over 600 E3 enzymes. At least 109 putative E3 genes have been identified in *T. cruzi* based on RING, Cullin, HECT, U-box or F-box domains (Gupta et al., 2018). However, detailed biochemical and structural studies on these proteins are largely lacking. Notwithstanding, a few studies reported cases of E3 ligases and their interacting partners in trypanosomes. For example, WD40-repeat protein-cullin RING E3 ligase (CLR^WDR1^) is expressed in *T. brucei* and controls the level of Polo-like Kinase (Hu et al., 2017). Moreover, Q4E398, Q386V9, and D0A601(Gupta et al., 2018) are atypical putative F-box ligases exclusively present in *Trypanosoma spp* and, therefore, may suggest their interaction with trypanosome-specific proteins. An increasing amount of evidence suggests the importance of the ubiquitin-proteasome pathway in maintaining the homeostasis of several essential proteins in trypanosomes (Bijlmakers, 2021; Gupta et al., 2018; Muñoz et al., 2015; Rojas et al., 2017) or trypanosomal proteins involved in drug susceptibility such as ISGs (Zoltner et al., 2015).

## 5. Introduction to the PROTAC technology

**Fig. 2.**
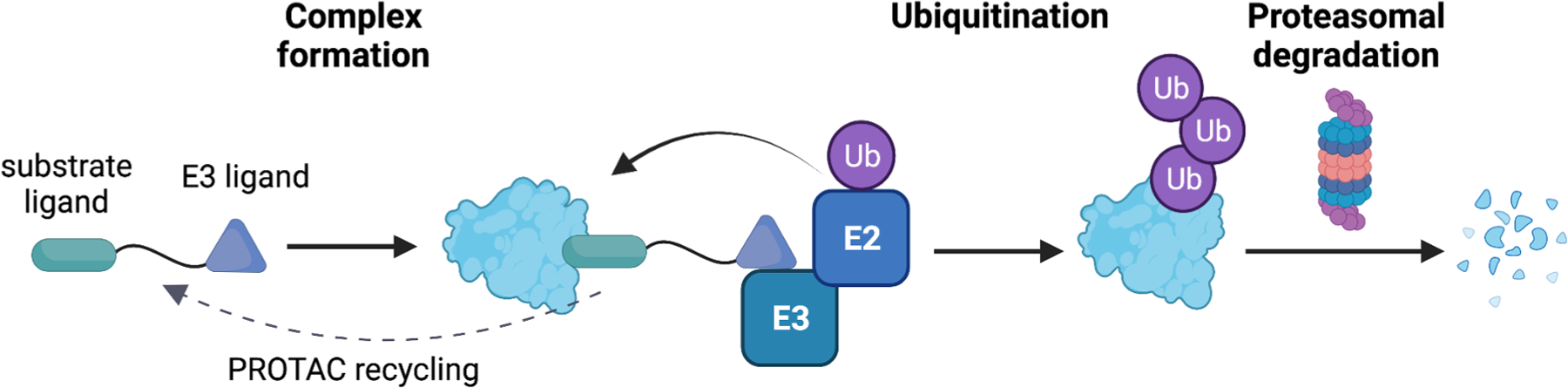
Mechanism of the PROTAC-mediated proteasomal degradation of a target protein. The process begins with recognition of E3 and the substrate protein by the PROTAC anchor and warhead ends, respectively. A complex of Ubiquitin-E2-E3-PROTAC-substrate is formed, which leads to the ubiquitination of the substrate. Ubiquitinated substrate is finally recognized and degraded by the 26S proteasome. *Figure Created with* BioRender.com.

Protein degradation is a natural cellular process of protein turnover (Schapira et al., 2019), and scientists have hijacked this process by developing the proteolysis-targeting chimeras (PROTACs; Fig. 2) to degrade proteins of interest (POI) involved in different types of diseases (Lai and Crews, 2017). PROTACs are heterobifunctional molecules consisting of two ligands connected covalently via a linker (Toure and Crews, 2016). They offer several advantages over conventional small molecules in many ways. Firstly, they facilitate the degradation of the POI, which removes also any of its scaffolding function. Moreover, their fast and irreversible action does not necessitate prolonged association of the drug with POI - PROTACs have become the lead examples of the event-driven rather than occupancy-driven pharmacology. This fast POI turnover helps circumvent drug resistance, which may arise due to the acquisition of mutations in some residues involved in POI interaction with the inhibitor. The emergence of such mutations may be facilitated by the scaffolding functions of the targeted protein or by some persisting activity of a portion of POI that could not be fully saturated by the classical inhibitors. PROTACs may therefore subvert the emergence of drug resistance, which is a very promising property not only in the treatment of cancer (Burke et al., 2022), but also would be invaluable against infectious diseases. Secondly, PROTACs exhibit high selectivity and can degrade proteins considered “undruggable” through conventional small-molecule inhibition since they require only the POI ligand and not a functional inhibitor (Edmondson et al., 2019; Lu et al., 2018). Owing to their ability to dissociate from the ubiquitinated POI and partake in the subsequent recruitment of another molecule of POI, a low, substoichiometric dose is required to bring about the degradation of the target protein (Sun et al., 2019). These features make them highly attractive therapeutic agents, and several PROTACs have been developed to degrade proteins involved in different cancers, neurodegenerative diseases, and immune disorders.

First-generation PROTACs were peptide-based, which were first reported by in 2001 by the Crews and Deshaies group to target methionine aminopeptidase 2 (Sakamoto et al., 2001). Though effective and still being developed (Ma et al., 2020), peptide-PROTACs’ major limitation is their micromolar activity range, probably due to their size and poor cell permeability. This shifted the focus of PROTAC development from peptides to small molecules. Since then, highly effective small-molecule PROTACs have been developed to target many clinically important proteins. Prominent examples include the estrogen and androgen receptors-degrading PROTACs currently undergoing clinical trials (Flanagan and Neklesa, 2019; Neklesa et al., 2019).

### 5.1. Design

PROTAC design involves the covalent joining of two ligands via a linker molecule. The ligand that recruits the substrate-binding domain (SBD) of an E3 ubiquitin ligase is the “anchor,” while the other ligand molecule, which binds to the POI, is known as the “warhead” (Burslem and Crews, 2017).

### 5.2. Anchor

PROTAC is still a maturing technology, with scientists still searching for different molecules to serve as anchors that can recruit different E3 ubiquitin ligases, since the choice of E3 could affect degradation efficiency and tissue specificity (Troup et al., 2020). The first reported PROTAC used IκBα phosphopeptide as an anchor to recruit F-box protein “β-transducin repeat-containing protein” (SCF^βTRCP^) E3 ligase complex (Sakamoto et al., 2001). However, the requirement for phosphorylation of IκBα phosphopeptide limits the application of this PROTAC. Schneekloth et al. (2008) made the first progress by using a 7-amino-acid peptide derived from hypoxia-inducible factor 1α protein (HIF-1α) ≈ a natural substrate of von Hippel–Lindau protein (VHL), as an anchor to recruit VHL E3 ligase. Although these PROTACs were successful in inducing protein degradation, they operate at relatively high concentrations due to their poor cell penetration and are prone to immune attacks (Li and Crews, 2022). Thereafter, remarkable improvements have been made with respect to designing small molecule VHL anchors and also recruiting other E3 ligases. The mouse double minute 2 homologue (MDM2), a cellular inhibitor of apoptosis protein 1 (cIAP1), and especially cereblon (CRBN) are distinguished examples of E3 ligases recruited by PROTACs, to a great extent by small-molecule anchors, for example derived from thalidomide which binds CRBN (Hines et al., 2019; Itoh et al., 2010; Winter et al., 2015). After the discovery of the thalidomide mode of action, CRBN became in particular exploited for TPD, with the highest representation in the earliest batch of PROTACs that entered clinical trials (Békés et al., 2022).

### 5.3. Warhead

Since PROTACs require only selective binding and not inhibition, different molecules have been explored as warheads of PROTAC machinery due to their ability to interact with the target protein. Like anchors, peptides have also been utilized as PROTAC warheads, and their design is relatively less tedious than small molecules. Peptide warheads are designed based on the structurally identified motifs of proteins interacting with the POI in question instead of the screening of large chemical libraries in search of small molecule warheads. Permeability of peptide PROTACs can be improved by including a cell-penetrating peptide motif (e.g. poly-Arginine), and an exciting example of such modified fully-peptide PROTAC is TH006 targeting Tau, which could decrease the cytotoxicity caused by Aβ in an Alzheimer’s disease mouse model (Chu et al., 2016). Phosphorylation sites on peptide PROTACs have also been exploited as a means of conditional degradation regulation, for example in the PI3K “Phospho-PROTAC” which provided the first evidence for in vivo anti-cancer activity of PROTACs in murine models (Hines et al., 2013). As long as a molecule can interact with the POI with a moderate affinity (K_D_ ≤1–500 nM) (Békés et al., 2022), and can retain this interaction when derivatized with a linker, its potential as a PROTAC warhead cannot be overruled. As small-molecule warhead examples, the earliest PROTAC designs used ovalicin, a covalent methionine aminopeptidase inhibitor (MetAP-2) (Sakamoto et al., 2001), or estradiol and dihydrotestosterone to target estrogen and androgen receptors, respectively (Sakamoto et al., 2003). A classical warhead example which is used to test new TPD approaches is JQ1, a pan-inhibitor of human Bromo- and Extra-Terminal (BET) proteins which binds their bromodomains with K_D_ ~100 nM (Raina et al., 2016; Zengerle et al., 2015). Known inhibitors employed as PROTAC warheads often gain higher selectivity, as research on kinase inhibitors has shown – as a remarkable example, foretinib could target different isoforms of the p38 MAPK kinase when coupled to VHL ligand using a different linker configuration (Smith et al., 2019). This raises hope than even promiscuous ligands or warheads against conserved targets can become more POI- and species-selective when employed in TrypPROTACs. Another class of molecules that are currently being explored as PROTAC warheads are nucleic acids (Békés et al., 2022), including aptamers. Aptamers are single-stranded oligonucleotides, typically less than 100 bases, that are derived through iterative selective evolution of ligands by exponential enrichment (SELEX), and their major advantage lies in their ability to discriminate between closely related targets with exceptional selectivity. Compared to small molecules or peptides, aptamers are synthesized through a less time-consuming and inexpensive process and are easier to manipulate (Ni et al., 2021). Studies have already shown the degradation of nucleolin by aptamer PROTACs, either using the AS1411 aptamer directly as a warhead or in a form of an Aptamer-PROTAC Conjugate (APC) which improved tumor selectivity of a BET PROTAC (Chen et al., 2023; He et al., 2021; Zhang et al., 2022), thereby underscoring their potential in TPD. However, nucleic acid-containing PROTACs as newcomers have not yet been represented in clinical trials and their clinical usefulness remains yet to be demonstrated beyond application as research tools.

### 5.4. Linkers

The PROTAC linker molecule serves as a bridge between the anchor and warhead ligands. It has been proven that the targeting selectivity and the overall efficiency of a PROTAC molecule is dependent not only on the anchor and warhead but also on the linkers (Gadd et al., 2017; Roy et al., 2019; Sun et al., 2019). A different linker attachment site may result in altered selectivity, as the example of foratinib-VHL PROTACs targeting different p38 MAPK isoforms has shown (Smith et al., 2019). The length and nature of the linker may also affect degradation efficiency and PROTAC stability. PROTAC “linkerology” is still however mostly a trial-and-error process requiring experimental validation. Historically, very few types of chemical motifs have been used as linkers. PEG (polyethylene glycol) and alkyl linkers are the most widely considered in most cases. However, a database has been developed by Maple et al. (2019) to provide information on the list of available PROTAC linker motifs. This database lists linker molecules, including PEG, alkyl, other glycols, alkyne, triazole, piperazine, and other moieties (Maple et al., 2019).

### 5.5. Mechanism of action

**Fig. 3.**
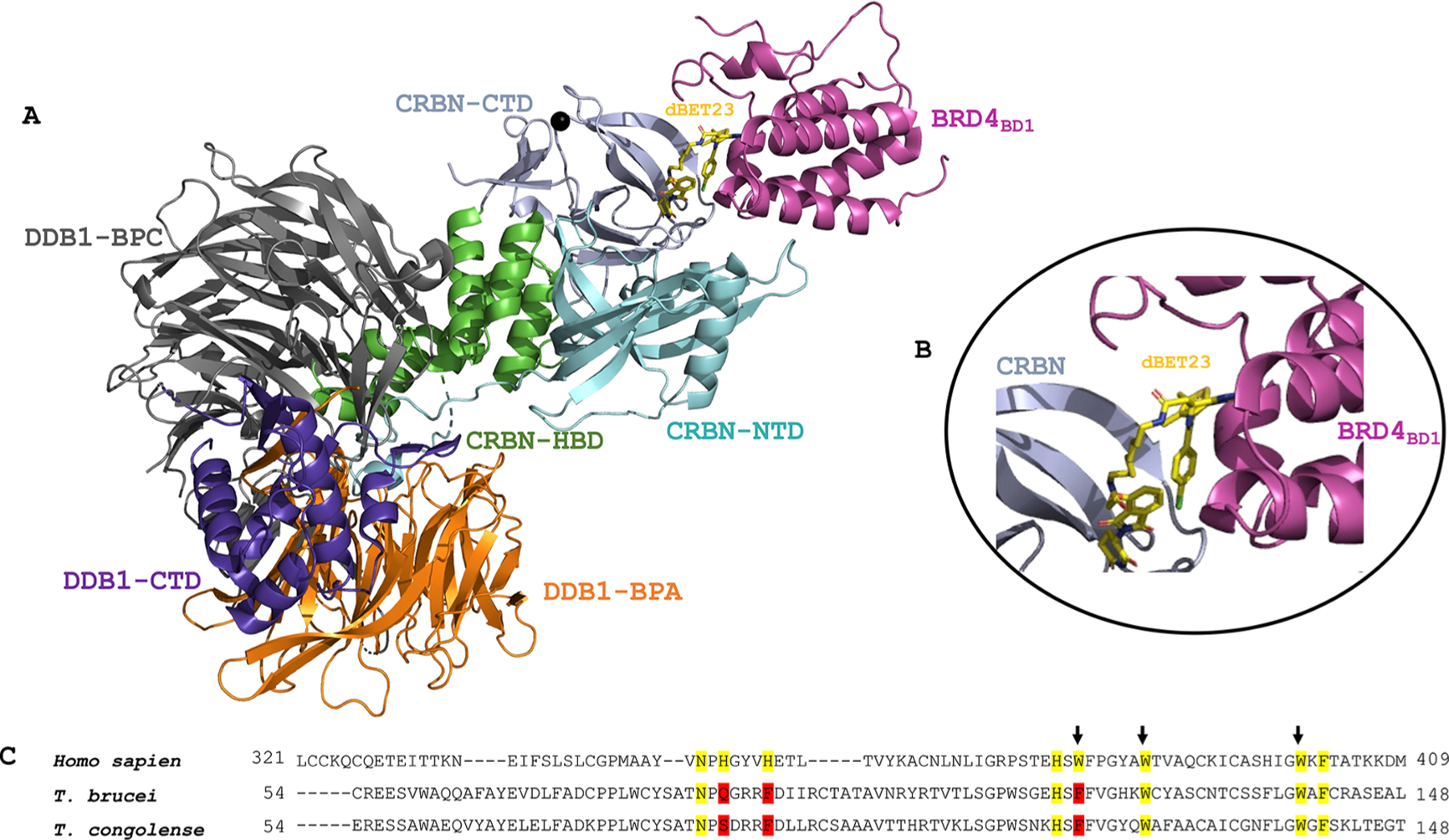
**(A)** Crystal structure of the human CRBN-dBET23-BRD4 ternary complex (PDB ID: 6BOY). The interaction is mediated by dBET23 PROTAC. (B) Enlarged view of the CRBN-dBET23-BRD4 ternary complex interaction interface. (C) Protein sequence alignment of human CRBN with *Trypanosoma* CULT-domain containing proteins. Thalidomide-binding residues are highlighted in yellow, while the tri-Trp-cage that forms the thalidomide-region is shown with arrows above, and residues from this subset which are not conserved in trypanosomes are indicated in red.

Although PROTAC is a relatively recent emerging technology, the multiple academic and industrial efforts have already yielded a substantial understanding of the mechanism of PROTAC action (Deshaies, 2015). Targeted protein degradation mediated by PROTACs is analogous to the cellular proteasomal-mediated protein degradation, a multistep and sequential process coupled with several enzymatic reactions (Thrower et al., 2000). E3 ligases are readily available in nature (more than 600 in humans), but the choice of E3 used may depend on the target protein of interest, especially in terms of cellular localization, tissue expression and ubiquitin linkage type, as not all ubiquitination linkage patterns may lead to degradation (Berndsen and Wolberger, 2014). – mostly the key Lys48- and Lys11-linked ubiquitin chains are being exploited, but for many ligases such information is still unknown. The PROTAC anchor binds to E3-E2-ubiquitin, and the warhead captures the target protein while the linker maintains the optimal distance between the E3 complex/anchor and the POI/warhead (Lai et al., 2016). In a crucial step, a ternary complex of POI-PROTAC-E3-E2-Ubiquitin is formed (Fig. 3A), facilitating the ubiquitination of the target protein (Churcher, 2018). The proteasome then degrades the (poly)ubiquitinated protein.

### 5.6. E3 ligases for targeted protein degradation in trypanosomes

Ubiquitin ligases (E3) are central to the functioning of UPS as they facilitate the transfer of ubiquitin to the target substrate. Cullin-domain-containing E3 ligases (Cullin–RING ubiquitin ligases, CLRs) are the largest family of E3 ligases, and most of the reported PROTACs leverage on ligases from this family for targeted protein degradation (TPD). The VHL and CRBN E3 ligases are the most utilized CLRs in the TPD field (Girardini et al., 2019). The availability of VHL and CRBN ligands with suitable physicochemical properties and well-studied binding poses makes these E3 ligases even more attractive for TPD (Ishida and Ciulli, 2020). As such, the utility of these ligases should be given due consideration in respect to TPD in trypanosomes. The genes of at least seven E3-ligases belonging to the CLRs family has been annotated in African trypanosome genomes. Among these, we found a CULT-domain containing protein homologous to human CRBN, conserving five of the eight residues of the thalidomide-binding region (UniProt, D0A9D4). Most importantly, two of the three tryptophan residues constituting the tri-Trp-cage that forms the thalidomide-binding region were also conserved in the *Trypanosoma* CULT-domain containing protein. The other Trp-residue was substituted for another aromatic residue, phenylalanine (Fig. 3B). Therefore, this protein represents an ideal starting candidate E3 ligase for designing thalidomide-based anti--trypanosomal PROTACs, though we envision that quite substantial ligand optimization would have to be done to match the trypanosomal homologue. Unfortunately, no homolog of VHL E3 ligase has been found in trypanosomes proteome, suggesting that human VHL ligands cannot be repurposed for trypanosomal TPD. Nonetheless, dozens of putative E3 ligases (e.g. 13 Cullin genes, 67 RING genes in *T. cruzi*) have been reported in trypanosomes (Gupta et al., 2018) which can be explored for anchor design, keeping in mind that their applicability in designing anti-trypanosomal PROTACs should be validated by in-depth biochemical and structural interrogation of these proteins. Once experimental structural models of trypanosomal E3 ligases become available, they in particular might direct the choice and design of E3s and their ligands. It is also worth mentioning that the predicted structures of a good number of trypanosomal ligases are available in the AlphaFold protein structure database (Jumper et al., 2021) and can be resourceful in PROTAC anchor design. Ideally, to decrease the chance of resistance emergence, the future TrypPROTAC design should exploit an E3 enzyme that is essential for the parasite. Here, RNAi or TPD research tools may come in handy to validate E3 candidates - as an example, the loss of Rbx1 (a component of Rbx/Cullin E3 complex) was reported to affect kinetoplast replication, but the knockdown of the related Cullin was neutral to the trypanosome (Rojas et al., 2017; Zoltner et al., 2015). Finally, the use of E3s specific for the parasite (Gupta et al., 2018) might help design more selective drugs with lower toxicity. Additionally, the use of both trypanosome-specific E3 ligands and warheads may ensure synergistic species selectivity. However, it is worth noting that even promiscuous warheads have gained selectivity upon conversion into PROTACs, and similarily even E3 ligands recruiting a universally conserved E3 ligase may become more selective in an optimized TrypPROTAC.

### 5.7. Potential PROTAC targets in african trypanosomes

One of the most admired properties of PROTACs is their ability to facilitate the degradation of both druggable and undruggable proteins. Nevertheless, our discussion here would only focus on proteins already confirmed to be druggable, with well-characterized interaction with their inhibitors and/or substrates. These inhibitors can serve as warheads of PROTACs that might degrade essential proteins of trypanosomes. Table 1 represents selected *T. brucei* proteins and a few of their experimentally determined ligands which may be considered for PROTAC design and targeted protein degradation.

### 5.8. Alternative oxidase and glycerol kinase

Trypanosoma alternative oxidase (TAO) is a cyanide-insensitive oxidase that catalyzes the oxidation of ubiquinol with concomitant reduction of oxygen to water. It is the only terminal oxidase in the respiratory chain of bloodstream-form (BSF) *T. brucei*. It serves the ultimate function of maintaining the NAD+/NADH balance needed for stable energy production through glycolysis. As glycolysis is the primary energy-generating pathway in BSF trypanosomes, coupled with the absence of a TAO homolog in the parasite’s mammalian host, TAO was identified as a suitable drug target for the treatment of AT (Chaudhuri et al., 2006; Hill, 1976). For example, the inhibition of TAO with ascofuranone is associated with a reduction in ATP production through glycolysis by half (Minagawa et al., 1996). Trypanosomes resolved this issue by conversion of glycerol 3-phosphate (G-3-P) to glycerol; a reaction catalyzed by trypanosomal glycerol kinase (TGK), which yields 1 ATP to compensate for the energy deficit as a result of TAO inhibition and at the same time restors the disrupted NAD+/NADH balance, at least for a while (Balogun et al., 2017). This implies that targeting only TAO is insufficient to compromise energy production in BSF. Interestingly, the reverse reaction of TGK prevails once glycerol accumulates. In fact, co-administration of ascofuranone with glycerol resulted in the instant elimination of trypanosomes from circulation in *T. vivax* infected mice (Yabu et al., 2006). However, the glycerol concentration needed (5–10 mM) for co-administration with ascofuranone is clinically impracticable. This concentration will equally inhibit human GK. Structural studies revealed that trypanosome GK differs from mammalian GK in functional regions and this allowed for the investigation of trypanosome-specific GK inhibitors that could be used for combination therapy (Balogun et al., 2014). In search of a TGK inhibitor, a recent study discovered a hydroxycoumarin derivative that potently inhibits both TGK and TAO (Balogun et al., 2019). The compound **k**ills trypanosomes both in the presence and absence of ascofuranone and is not toxic against the human cell line, suggesting a lack of interaction with human GK. Therefore, the hydroxycoumarin derivative could be employed as a warhead to attract both TGK and TAO for ubiquitination and subsequent proteolysis.

### 5.10. Cysteine proteases

Cysteine proteases are expressed in the entire life cycle of the trypanosomatids. They are known to participate in the parasites’ essential cellular processes such as nutrition, reproduction, invasion of host cells, and escape of the host cell immune system (Lecaille et al., 2002). Due to their pivotal role in the parasite’s life cycle, these papain-like proteases are actively involved in the onset and progression of African trypanosomiasis and Chagas disease (Turk et al., 2012). As a result of a better understanding of the role of these enzymes in disease physiopathology, inhibition of cysteine proteases by small-molecule compounds has been proven to be a practical strategy in drug discovery for the parasitic infection (Kerr et al., 2010; Martinez-Mayorga et al., 2015; Steverding et al., 2012). *Trypanosoma brucei* expresses two major lysosomal cysteine proteases belonging to the papain family, the cathepsin B-like *T. brucei* Cathepsin B (TbCathB) and the cathepsin l-like protease rhodesain (Steverding et al., 2020). While the expression of TbCathB varies strongly during the life cycle of the parasite, rhodesain is constitutively expressed (Mackey et al., 2004). During the disease progression into the second stage, rhodesain is involved in crossing of the parasites into the central nervous system via the host’s blood-brain barrier (Grab et al., 2009; Nikolskaia et al., 2008). According to Alsford et al. (2014), inhibition of rhodesain and not TbCathB led to cessation in the parasites’ growth and an increased sensibility to lytic factors in human serum, thereby confirming the essential role of rhodesain in parasite survival, proliferation, and pathogenicity. Consequently, rhodesain is considered the main lysosomal cysteine protease in trypanosomes and presents a promising anti-trypanosomal drug target (Schirmeister et al., 2016). Inhibitors of rhodesain, including peptides, natural compounds, and synthetic small molecules, have been explored (Ettari et al., 2013; Jefferson et al., 2016; McShan et al., 2015). However, structural resemblance to host proteins poses problems with off-target effects since rhodesain shares about 50% sequence identity with human cathepsin L (hsCathL). Nevertheless, selective peptidomimetic compounds that target only rhodesain were reported to inhibit the enzyme at a low micromolar range and are inactive against human cathepsin L (Ettari et al., 2016). Among this series, (R, S, S)–3 proves to be the most effective, and its utilization as a warhead could be considered in the development of PROTACs for rhodesain degradation.

### 5.11. Glyceraldehyde-3-phosphate dehydrogenase

The glyceraldehyde-3-phosphate dehydrogenase (GAPDH) is a glycolytic enzyme that catalyzes the conversion of glyceraldehyde 3-phosphate to 1,3-bisphosphoglycerate in the sixth step of the glycolysis. As glycolysis is the primary energy source in the bloodstream form *T. brucei,* inhibition of TbGAPDH and other rate-limiting enzymes of glycolysis abolish energy production in trypanosomes, which makes them attractive drug targets for the treatment of AT (Zhang et al., 2013). Most of the inhibitors of TbGAPDH were discovered 20–30 years ago, and many of them have been proven to be less effective and, to some extent, toxic to humans (Barnard et al., 1993; Duszenko and Mecke, 1986). Belluti et al. (2014) developed a series of quinone–coumarin hybrids that can inhibit Glyceraldehyde-3-phosphate dehydrogenase and trypanothione reductase. Among this series, an integrated derivative 2-{4-[6-(2-*dimethylaminoethoxy*)-2-*oxo*-2*H*-*chromen*-3-*yl*]phenoxy} anthracene-1,4-dione shows remarkable efficacy against *T. brucei* with negligible toxicity to mammalian cells (Belluti et al., 2014). It is unfortunate to say that this was the only progress made in searching for inhibitors of TbGAPDH in the recent years. While the derivative mentioned above may be considered in designing warheads, there is a need to explore more chemical scaffolds to identify highly selective TbGAPDH inhibitors. This will allow the maximization of the potential of TbGAPDH as a target for targeted proteolysis in African trypanosomes.

### 5.12. Glycogen synthase kinase-3

Glycogen synthase kinase-3 (GSk-3) is a serine-threonine kinase that regulates glycogen synthase activity through phosphorylation (Parker et al., 1983). GSK-3 acts on glycogen synthase and phosphorylates other substrates, making the enzyme crucial in a number of cell-signaling pathways. GSK-3 is widely distributed among eukaryotes, with homologs present in yeast, mold, protozoans, plants, and mammals (Droucheau et al., 2004; Meijer et al., 2004; Plyte et al., 1992; Xingi et al., 2009), irrespective of whether they require glycogen or glycogen synthase. Owing to the role of GSK-3 in many physiological processes, inhibition of this enzyme has been demonstrated to be attractive in treating diseases like Alzheimer’s dementia and diabetes mellitus (Bhat et al., 2004; Ring et al., 2003). Relevant to our discussion, *GSK-3* knockdown in *T. brucei* is lethal to the parasite, and inhibitors of GSK-3 are generally trypanocidal (Ojo et al., 2008). Therefore, GSK-3 is a validated therapeutic target for the treatment of African trypanosomiasis. Inhibitors of GSK-3, like indirubins, often target the conserved ATP binding site of the enzyme, and as such, selectively of inhibitors of GSK-3 against human kinases is the most significant bottleneck to this therapeutic approach (Knockaert et al., 2004; Leclerc et al., 2001; Short and Growth, 2019). However, finding a trypanosome-specific GSK-3 inhibitor is more than possible since it shares only 43.5% homology with human GSK-3 (Ojo et al., 2011). An attempt to optimize the selectivity of aminopyrazole derivatives resulted in compound 9 g (Table 1, S/N: 3), which potently inhibits TbGSK-3 and is selective against at least 124 human kinases. Unfortunately, 9 g strongly inhibits (>90%) human GSK-3 at 10 μM concentration (Urich et al., 2014). Another structure-activity relationship study revealed a pyrazolo[1,5-b] pyridazine derivative (20 g) as a highly selective inhibitor of *T. brucei* GSK-3, though 20 g (Table 1, S/N: 3) was found to be toxic in mice which limits its applicability (Tear et al., 2020). Moreover, the selectivity of 20 g was only tested against three human kinases; GSK-3, cyclin-dependent kinase-2, and cyclin-dependent kinase-4. Therefore, it is unclear whether this compound would display off-target binding to other human kinases. To take advantage of TbGSK-3 as a therapeutic target for the treatment of AT, there is a need to revisit the selectivity of its inhibitors. Optimizing TbGSK-3 ligands to discriminate against human kinases will be crucial in using this enzyme for targeted proteolysis.

### 5.13. Triose-phosphate isomerase

Triosephosphate isomerase catalyzes the fifth step of the glycolytic pathway, which involves the interconversion of glyceraldehyde 3-phosphate and dihydroxyacetone phosphate (Galland et al., 2010). Structurally, triosephosphate isomerase from *T. brucei* (TbTIM) is a homodimeric enzyme constituting 250 amino acid-residues monomeric units, and the enzyme is only active in its dimeric form (Schnackerz et al., 1991). Targeting TbTIM dimerization interface with small molecules or peptides can incite structural modifications leading to enzyme inactivation. Indeed, benzimidazole derivatives were confirmed to disrupt this intercept and inhibited only the TbTIM and not human triosephosphate isomerase (Vázquez-Raygoza et al., 2017). Although an attempt to target this interface with peptides was not successful, a lysine-dependent inhibition of TbTIM by a group of hydrophobic peptides was reported (Kuntz et al., 1992). These peptides can discriminate between TbTIM and triosephosphate isomerase from rabbit muscle, yeast, and *Escherichia coli*, implying their high selectivity. Garza-Ramos et al. (1998) reported other small molecule inhibitors of TbTIM, such as dithionitrobenzoic acid and methylmethane thiosulfonate. Yet, the latter compounds are not specific and would not be relevant to our subject matter. Like other drug targets in trypanosomes, not many TbTIM inhibitors have been discovered, majorly due to lack of funding and incentives. However, the benzimidazole derivatives and the hydrophobic peptides reported by Kuntz et al. (1992) could be useful in PROTAC warhead design to target TbTIM.

### 5.14. Trypanothione reductase

Trypanothione reductase (TR) is a flavoenzyme unique to trypanosomes and Leishmania. It catalyzes the NADPH-dependent reduction of oxidized trypanothione (T[S]_2_) in an analogous way to the reduction of glutathione by human glutathione reductase (GR). This system checks the ROS level in trypanosomes, and TR and its substrate considerably differ from human GR and glutathione (GSSG). More importantly, both TR and GR are highly specific enzymes and do not cross-process each other’s substrates. TR is a validated drug target by virtue of compromising the redox defense of the parasite when the enzyme is inhibited. Many inhibitors of TR have been identified with structural information for at least 21 different ligands in complex with TR (Battista et al., 2020). However, most of these inhibitors bind to the mepacrine binding site of TR, which happens to be very broad and quite promiscuous. This has resulted in the low potency of these ligands against TR and poor selectivity against human GR and thioredoxin reductase. Recently, an imidazole-phenyl-thiazole derivative **3f** (Table 1, S/N: 6) was found to prevent the dimerization of *Leishmania infantum* trypanothione reductase monomers and inhibit its enzymatic activity by binding to the dimerization interface of the enzyme. Molecular dynamics studies revealed that compound **3f** interacts with Asp432, Ile437, Val460, Glu466, and Phe454 at the interface (Revuelto et al., 2019), and protein BLAST has confirmed the conservation of these residues in *T. brucei* trypanothione reductase, suggesting the potential applicability of **3f** in African trypanosomes. On these grounds, **3f** may represent a starting point for the design of PROTAC warhead to target trypanothione reductase.

### 5.15. Ornithine decarboxylase

The first step of the polyamine biosynthetic pathway in *T. brucei* is catalyzed by ornithine decarboxylase (Phillips, 2018; Kim et al., 2017). Ornithine decarboxylase (ODC) catalyzes the conversion of ornithine to putrescine which is necessary for cell replication in *T. brucei* (Macedo et al., 2017). On that account, ODC is a promising drug target for treating AT. Eflornithine (α-difluoromethylornithine or DFMO) is a substrate analog and an inhibitor of ODC (LoGiudice et al., 2018) and has been used for more than 50 years for the treatment of HAT. However, as we highlighted earlier, Eflornithine is toxic and only effective in late-stage treatment for HAT. Moreover, Eflornithine is also ineffective against parasites with high ODC turnover like *T. b. rhodesiense* (Carrillo et al., 2000). The search for ODC inhibitors is partly limited by the lack of a simple assay method to monitor the enzyme’s activity. The available methods rely on radiolabeled substrates, which are not practical for high throughput screening (Bitonti et al., 1982). Smithson et al. (2010) reported four chemotypes that are potent and effective inhibitors of trypanosomes ODC; bisbiguanide, benzothiazoles, indoles, and dithioamidines. Even so, only dithioamidines among these chemotypes were selective against human ODC (Smithson et al., 2010). Thus, dithioamidines could be relevant in targeting *T. brucei* ODC for proteolysis.

### 5.16. UDP-Galactose 4′ epimerase

Galactose metabolism plays a pivotal role in ensuring trypanosome infectivity. The transferrin receptor and VSG molecules are galactose-containing glycoproteins central to the parasite’s survival in the mammalian host. The former facilitates the uptake of iron from the host to synthesize heme, and the latter ensures host immune evasion (Alphey et al., 2006). Trypanosomes lack transporters to uptake galactose from extracellular environments, and as a result, they meet their galactose requirement by the epimerization of glucose to galactose. *Trypanosoma brucei* UDP-Galactose 4′ epimerase (TbGalE) is the enzyme in charge of such interconversion in African trypanosomes (Shaw et al., 2003) and its reaction is essential to maintain the parasite’s virulence and hence viability (Roper et al., 2005, 2002). Despite the principal role of TbGalE, there are not many drug discovery programs targeting this protein, and some of the discovered inhibitors are toxic to human cells (Urbaniak et al., 2006). Notwithstanding, Durrant et al. (2010) discovered 2′- carbamoyl-[1,1′-*biphenyl*]–2-carboxylate derivatives that inhibit TbGalE in vitro but failed to kill cultured trypanosomes. This was explained by the hydrophobicity of the molecules, considering that the less lipophilic compound among them (clorobiocin) was trypanocidal. It is tempting to speculate that the pharmacokinetics of these compounds may be improved when they are integrated into a PROTAC molecule. Be that as it may, the selectivity of this series is not documented, so it is unclear whether they will equally bind human GalE. However, since the deficiency of human GalE is either asymptomatic or causes an uncommon type of galactosemia that can be partly regulated by diet and well-tolerated for a brief duration, such selectivity may not be required (Hamosh et al., 2002). If this happens to be the case, even TbGalE substrate analog UDP-4-deoxy-4-fluorogalactose may be considered in PROTAC design as inhibition is not required for PROTACs to elicit their function.

## 6. Targeted protein degradation beyond the classical PROTAC approach

The PROTAC technology has, so far, been proven to be successful in targeting proteins for proteasomal degradation and has in recent times become a powerful therapeutic strategy for managing many diseases. Having said that, issues such as the relatively large size of PROTACs, which affects their bioavailability, and the need to target E3 ligases themselves necessitated some improvements in this technology and the birth of new targeted protein degradation approaches. We herein discuss such alternative TPD approaches and their caveats, and the ways in which they can be applied to trypanosomal targets.

### 6.1. HomoPROTACS

The concept of HomoPROTACs stems from the idea that many E3 ligases themselves are essential for eukaryotic cell viability, as they serve vital roles in protein quality control, and their dysregulation often translates into a disease state. Thus, the inhibition or degradation of E3 ligases is desired in some pathological conditions. HomoPROTACs are designed to contain two ligands of the same E3 ligase connected by a linker, and the goal is to attract two molecules of that same E3 ligase. The binding of HomoPROTAC to its target brings about cross-ubiquitination of the bound E3 ligases and their subsequent degradation. Prominent examples of HomoPROTACs include the ones designed to target VHL and CRBN E3 ligases (Maniaci et al., 2017; Steinebach et al., 2018). Similarly, E3 ligases are equally essential to the pathogenesis and survival of trypanosomes (del Pino, 2020; Gupta et al., 2018). Gupta and co-workers have highlighted some atypical E3-ligases specific only to trypanosome species (Gupta et al., 2018). Therefore, we propose that efforts should be intensified to unveil the cellular role of these atypical trypanosomal ligases and their suitability as targets of HomoPROTACs.

### 6.2. PHOTACs

PHOtochemically TArgeting Chimeras (PHOTACs) are a special type of PROTACs activated by light. They comprise a ligand of POI, a photoswitch, and the ligand of an E3 ligase. The first PHOTACs were reported by Pfaff et al. (2019), which they used to target proteins from the bromodomain and extraterminal domain (BET) family. The major advantage of PHOTACs over classical PROTACs is the ability to regulate their activity with spatiotemporal precision. Although PHOTACs are presumed to be more powerful as research tools in cell biology studies, it is speculated that they could as well be applicable in clinical practice (Reynders et al., 2020). One way this could be achieved is by activating them with light prior to administration with the purpose of limiting their activity to a short period of time. This could be useful in designing trypanosomal proteins degraders that may cross-react with human proteins, for example, if the ligands of human CRBN are repurposed for anti-trypanosomal HomoPROTACs. Here, the rate of the inactivation of the photoswitch can be capitalized on to minimize the undesired degradation of Human CRBN.

### 6.3. SNIPERs

Specific and Non-genetic IAP-dependent proteins Erasers (SNIPERs) are molecules that capitalize on the E3 ligase activity of human inhibitor of apoptosis proteins (IAPs). SNIPERs differ from classical PROTACs by their ability to facilitate the ubiquitination of E3 themselves along with the target protein (Naito et al., 2019; Ohoka et al., 2017). As their name implies, IAPs overexpression is associated with decreased cellular apoptosis and cancer progression. Consequently, their downregulation is a promising approach to cancer therapy (Ma et al., 2021; Wright and Duckett, 2005). The absence of IAP homologs in trypanosomatids makes the SNIPER anchors irrelevant to our discussion, but an analogous approach could be beneficial if another class of E3 ligases acting as cell survival factors was identified in trypanosomes.

### 6.4. AUTACs

Autophagy-targeting chimeras (AUTACs) are special degraders that capitalize on the macroautophagy system, a mechanism by which cytoplasmic content such as protein aggregates are degraded through the fusion of the autophagosome with lysosomes (Feng et al., 2014). An AUTAC system consists of a degradation tag (S-guanine) linked with a ligand that recruits the target protein or organelle. The interaction of AUTAC with its substrate causes the ubiquitination of the substrate, resulting in phagocytosis of the target within a double-membraned phagophore (Takahashi et al., 2019). This system was developed to address the inability of PROTACs to induce the degradation of intracellular proteins that are not substrates for proteasomal degradation (Takahashi et al., 2019). Autophagy is a conserved process among most eukaryotes (Feng et al., 2014), and around half of the components of this system was reported to be found in trypanosomes, though with low sequence conservation (Brennand et al., 2012; Li et al., 2012). However, trypanosomes have complex and unique biology of membrane-bound organelles, as exemplified by the glycosomes – specialised peroxisome-like organelles that sequester glycolytic enzymes in bloodstream forms, and which might be regulated by autophagy in a stage-and environment-dependent manner (Brennand et al., 2012). Autophagy and its components might therefore function in a differently regulated way in trypanosomes. Once this complex organelle biology is better elucidated, the AUTAC strategy might become exploited also in these parasites.

### 6.5. LYTACs

Lysosome-targeting chimeras (LYTACs) are similar to AUTACs but target extracellular and membrane-bound proteins. They utilize mannose-6-phosphate as an anchor to engage cation-independent mannose-6-phosphate receptor (CI-M6PR) for lysosomal targeting of extracellular or membrane proteins (Banik et al., 2020). Following the binding of LYTACs to CI-M6PR and the target protein, the complex is engulfed by the cell membrane, and a transport vesicle is created. Subsequently, CI-M6PR guides the translocation of the vesicle to the lysosome and the degradation of the target protein. Finally, the LYTAC molecule and CI-M6PR are recycled to the cell surface for another round of target protein recruitment (Banik et al., 2020). The application of LYTACs was shown in the degradation of extracellular and membrane proteins, including apolipoprotein E4, epidermal growth factor receptor, CD71, and programmed death-ligand 1 (Banik et al., 2020). In trypanosomes, lysosomal trafficking does not depend on the mannose-6-phosphate signal. The parasite genome does not encode the gene for CI-M6PR, suggesting that this type of system is lacking in the organism. However, a dileucine signal has been identified to be solely sufficient for targeting p67 (a lysosomal hydrolase) to *T. brucei* lysosome, although the signal receptor is still a puzzle yet to be solved (Allen et al., 2007). Notwithstanding, the dileucine peptide could be critical in probing targets for LYTAC-induced lysosomal degradation. Another frontier on which LYTACs can be applied in trypanosomes is by engaging the host CI-M6PR to target extracellularly-exposed parasite’s membrane proteins. Here, the chimera will consist of mannose 6-phosphate conjugated with a ligand of the trypanosomal membrane protein and will function to induce the delivery of the target to the host’s lysosomes. However, this approach could be challenging to realize since trypanosomal surface proteins are largely protected by VSG.

### 6.6. CLIPTACs

The in-cell click-formed PROTACs (CLIPTACs) methodology involves the *in-vivo* synthesis of PROTACs molecules from separately administered small-molecules warheads (Tomoshige and Ishikawa, 2021). This methodology was first established by the research group of Astex Pharmaceuticals, Inc., a subsidiary of Otsuka Pharmaceutical Co. Ltd. The primary purpose of CLIPTACs technology is to overcome the bioavailability and poor cellular penetration of conventional PROTACs. A CLIPTAC is synthesized using the catalyst-free electron demand Diels-Alder cycloaddition (IEDDA) to connect tetrazine (Tz)-tagged warhead for the target protein and *trans*-cyclooctene (TCO)-tagged E3 ligase ligand. As a proof-of-concept, Lebraud et al. (2016) employed Tz-thalidomide-based CLIPTACs to show successful degradation of two oncogenic targets, i.e., bromodomain-containing protein 4 (BRD4) and extracellular signal-regulated protein kinase (ERK1/2). The CLIPTAC approach holds promise in addressing the issue of bioavailability of the protein degraders, which will be an expected issue also in trypanosomal PROTAC development.

### 6.7. Direct proteasome recruitment via CIDEs

Chemical inducers of degradation (CIDEs) are chimeric compounds analogous to PROTACs, but which directly recruit the 26S proteasome instead of an E3 enzyme (Bashore et al., 2022). A macrocycle that binds 26S was linked to a POI ligand to effectively knock down the target protein, circumventing the need either for an E3 ligand or for exposed lysine residues on the target. The latter might be especially crucial for lysine-less proteins, which are enriched among UPS components (Szulc et al., 2023). CIDEs based on ligands of trypanosomal 26S might facilitate alternative approaches to the development of trypanocidal degraders, which might be especially useful if working E3 ligands cannot be identified.

### 6.8. Molecular glues

Unlike PROTACs, molecular glues (MGs) are monovalent small molecules that induce interactions of two proteins by promoting cooperativity. In the context of our discussion, an MG is usually a ligand of E3 ligase that, through its binding, remodels or complements the surface of the E3 to induce a novel protein-protein interaction with POI. The MG may interact only poorly with either E3 or POI by itself, but the tertiary E3-MG-POI complex is formed with a high affinity. Upon interaction, POI is ubiquinated by the E3 ligase and marked for proteasomal destruction (Kozicka and Thomä, 2021). The small sizes of MGs make them more desirable as drugs as they are more easily transported into the cell. However, their design is not trivial since it is heavily dependent on structural information of the desired ternary complex. Not surprisingly, most of the reported MGs were discovered serendipitously (Dong et al., 2021). Therefore, the future discovery and application of MGs would rely on the development of a rational screening method for these glues in trypanosomes. The work of Mayor-Ruiz et al. (2020) is setting the pace in this direction.

### 6.9. Hydrophobic tagging

Hydrophobic tagging (HyTag) mimics protein misfolding. It is based on the idea that adding a hydrophobic group to the surface of a target protein can mimic an unfolded protein appearance (such as exposure of a hydrophobic degron), hence leading to the POI degradation. The HyTag system is also based on a bifunctional compound: the ligand of the target protein is attached to the hydrophobic moieties, which are then recognized by the unfolded protein response (UPR), hence promoting proteasomal degradation. The first generation of HyTags were designed to target HaloTag fusion proteins using adamantyl–chloroalkane-linked ligands (Neklesa et al., 2011). Other hydrophobic groups used in the HyTag system include tertbutyl carbamate-protected arginine (Boc3Arg), benzylic, tricyclic, and cyclohexyl bulky groups (Neklesa et al., 2011; Shi et al., 2016). Choi et al. (2021) applied adamantyl-based HyTag to target steroid receptor coactivator-1 (SRC-1). This degrader successfully induced the degradation of SRC-1 and inhibited cancer metastasis. The HyTag technology differs from other “tag-based TPD systems” that require fusion of a tag with POI, which we describe below. Since the “tag” is appended by binding of a stand-alone degrader molecule, HyTags have the potential to be developed as a therapeutic approach. The merit of HyTag over other TPD systems is the absence of the requirement for an E3 ligase ligand, which presents an excellent opportunity in trypanosomes, considering the little information on the E3 repertoire in the parasite.

### 6.10. Tag-based degraders

Many flavours of targeted protein degradation agents are used for research purposes, to study protein function and to validate drug targets. Some of these approaches will be valuable tools for the establishment of the TPD field and drugs in trypanosomes, and we shortly review some of the popular systems, including those regulated by small molecules. Many of these research tools rely on the fusion of POI with a tag, which in trypanosomes is largely achievable, as demonstrated by the TrypTag resource in which fluorescent protein tags could be attached to 89% of the *T. brucei* proteome (Billington et al., 2023). Although a word of caution comes from anti-cancer PROTAC development, in which a candidate PROTAC may be effective against a tagged, fusion POI, but not its unmodified version, perhaps due to different geometry or exposure of additional surface/polypeptide stretch that may facilitate ubiquitination and degradation. Tagged POI approaches are therefore useful for validation of targets, but final drug development requires testing an untagged POI for degrader validation.

#### 6.10.1. dTAG system

The degradation tag (dTAG) system requires mutant FKBP-type peptidyl-prolyl cis-trans isomerase (FKBP12), such as FKBP12^F36V^, expressed in-frame with the target POI, where the fusion of the POI with FKBP12^F36V^ can be achieved by transgene expression or CRISPR-mediated locus-specific knock-in. Then a heterobifunctional degrader (designed from ligands of FKBP12F36V and E3-ligase, such as CRBN) is used to recruit the FKBP12F36V-POI to CRBN to facilitate ubiquitination and proteasomal degradation of FKBP12F36V-POI (Nabet et al., 2018). This system was designed for studying the immediate effect of protein loss.

#### 6.10.2. SMAShTag system

The small-molecule assisted-shut-off (SMASh) system involves tagging POI with hepatitis C virus protease NS3 and degron (degradation-inducing signals) via genetic modification. Upon translation, the NS3 protease cleaves itself together with the degron, leaving a fully functional POI. When the protease inhibitor asunaprevir is administered, NS3 and degron are retained together with the POI. The degron, therefore, induces the proteasomal and/or autophagosomal-lysosomal degradation of the whole complex (Chung et al., 2015). Like dTAG, SMAShTag is a powerful research tool for the controllable regulation of protein expression.

#### 6.10.3. Auxin-inducible degron

The auxin-inducible degron system relies on the interaction of plant Transport Inhibitor Response 1 protein (TIR1) with Auxin/Indole-3-Acetic Acid (Aux/IAA) protein family, which is facilitated by the plant hormone auxin. To apply this for TPD, TIR1 is introduced to the target eukaryotic cells, and the protein of interest is tagged with an IAA auxin-inducible-degron (AID). Treatment of these cells with auxin induces the interaction of TIR1 with AID. TIR1 then recruits the evolutionary conserved SCF (Skp1, Cullin, and F-box) E3 ligase complexes to ubiquitinate the POI for proteasomal degradation (Nishimura et al., 2009). Auxin-inducible degron is a very efficient system for studying protein function and can be useful in validating drug targets for TPD in trypanosomes.

#### 6.10.4. Nanobody/Intrabody-directed protein degradation

This system harnesses the power of antigen-antibody interaction to direct the POI (i.e., the antigen) to the proteasome for degradation. Nanobodies are single domain antibodies that can be raised against any POI via immunization of camelids followed by phage display to enable the selection of nanobodies with desired properties (Muyldermans, 2013). In a similar way to Auxin-inducible degron, nanobody mediated protein degradation requires the genetic fusion of nanobodies raised against the target POI to F-box protein of the SCF E3 ligase complex and their intracellular expression (intrabodies). The intrabody-POI interaction then allows the ubiquitination of the POI followed by its proteasomal degradation (Shin et al., 2015). This TPD approach is equally a research tool for studying the effect of protein depletion and has been applied to both mammalian and plant proteins (Baudisch et al., 2018; Shin et al., 2015). A recent work by Broster Reix et al. (2021) reported targeting of *T. brucei* cytoskeletal protein TbBILBO1 using intrabodies, which affected biogenesis of a cytoskeletal structure leading to rapid cell death. This study suggests that the intrabody-mediated degradation can be as well applied for validating TPD targets in trypanosomes, and offers TbBILBO1 as a validated protein target for which a nanobody is already available.

#### 6.10.5. deGradFP

As a proof of concept, a group from Oxford University has recently reported the efficient degradation of kinetoplastid kinetochore protein from *T. brucei* using the degradFP system, which relies on SCF E3 ubiquitin ligase complex to target GFP-tagged proteins via an engineered fusion of an F-box protein with a nanobody against GFP (Ishii and Akiyoshi, 2022). This is the first evidence that a tag-based TPD system is a working strategy in trypanosomes.

## 7. Conclusions

African trypanosomiasis is a neglected tropical disease that is still of significant public health concern in sub-Saharan Africa. Although the number of reported human cases has decreased rapidly in recent years, the battle against HAT is still not over due to increasing evidence of animals hosting human-infective trypanosomes and vice versa (Desquesnes et al., 2016; Wardhana and Savitri, 2018; Welburn et al., 2015). Therefore, a collective effort toward eradicating human and animal infections is necessary as it is difficult to predict when this emerging zoonosis can result in another epidemic. Vaccine design remains the most desired approach, but trypanosomes have devised a number of ways to undermine all efforts toward vaccine development, though there has been remarkable progress recently in the animal experimentation of invariant flagellum antigen from *T. vivax* (Autheman et al., 2021). The few small molecule inhibitors currently used for managing African trypanosomiasis suffer from several drawbacks. Most trypanocidal drug discovery efforts still focus on inhibitors that operate using occupancy-driven pharmacology as their mode of action (MOA). However, this does not apply to targets that lack enzymatic activity, such as the scaffolding proteins or proteins that operate through the protein-protein interaction (PPI) mechanism (Toure and Crews, 2016). The efficacy of drugs that act using the occupancy-driven MOA is strongly related to high drug doses, which often give room to increased drug side effects resulting from off-target binding (Adjei, 2006). Moreover, drug resistance is another bottleneck limiting the efficacy of the occupancy-deriving therapeutics (Holohan et al., 2013). To avoid resistance emergence and modulate targets that do not operate using the occupancy-driven mechanism, new therapeutic agents are required for managing African trypanosomiasis. PROTACs are such molecules that prove to address those shortcomings by inducing the proteasomal degradation of POI at relatively small doses (Blair et al., 2015; Coleman and Crews, 2018). They have successfully been used to suppress targets like ERRα, BRD4, androgen receptor, and estrogen receptor. Although most of the success stories of PROTACs were reported for different human cancers, there is a growing interest in the application of this technology to infectious diseases (Grohmann et al., 2022; Izert et al., 2021; Morreale et al., 2022; Powell et al., 2021). The chief machinery of protein degradation, i.e., the UPS system, operates in trypanosomes and is involved in the homeostasis of several indispensable proteins.

We reviewed the state-of-the-art knowledge on this system in trypanosomes with an emphasis on E3 ligases that could be suitable for the TPD of drug targets in the parasites. We further discussed many essential proteins crucial for trypanosome survival, including their ligands, that could be considered for targeted protein degradation. However, our list is not exhaustive, as there are several other essential proteins in trypanosomes that have been discussed elsewhere (Altamura et al., 2020; Cullen and Mocerino, 2017). Our choice of these targets is based on the availability of their 3D structures; we believe this information will be crucial for designing highly selective warhead ligands that would recruit only the parasite’s protein and avoid off-target effects. It is worth noting that the required selectivity of both anchor and warhead ends should ensure that the resulting PROTAC is highly specific for trypanosomal targets, surpassing the starting warhead. Most of the discussed targets lack active drug discovery programs due to a limited access to funding and a lack of incentives from the industry. Notwithstanding, the few selective ligands we described above could represent starting scaffolds for designing efficient degraders warheads for targeted protein degradation in African trypanosomes.

We intend to present our work as a blueprint to facilitate the realization of TPD in African trypanosomes. The recent demonstration of the degradFP system in *T. brucei* (Ishii and Akiyoshi, 2022) has substantiated our position that TPD is indeed attainable in trypanosomes. Be that as it may, the future development and application of PROTACs and related TPDs in AT may be challenging for various reasons. As the first specific quandary, most of the TPD approaches heavily depend on the ability of the anchor compound to recruit the appropriate E3 ubiquitin ligase. To design the ideal anchor, the biochemical and structural information on the E3 ligases repertoire in trypanosomes (currently lacking) and their interacting ligands or partners needs to be unraveled. However, the CIDEs and HyTag systems (which have no requirement for E3 ligase) should, in principle, be relatively less complicated and could be ideal for pursuing TPD in trypanosomes. Other degraders like LYTACs which target membrane proteins, could be shielded from their targets by VSG molecules on the surface of trypanosomes and consequently more difficult to realize. We deemed the concept of aptamer PROTACs promising with respect to the design of warheads or even anchors, since like antibodies, they can be raised against almost any targets in a cheap, fast, and more efficient manner; thus, extending the capacity of PROTACs in targeting both enzymatic and non-enzymatic drug targets. However, it is unclear whether nucleic acid conjugates would have a better chance in delivery to the trypanosomal cytoplasm than classical PROTACs – given the higher electrostatic charge such compounds would be more soluble but less membrane permeable, and likely would require hijacking an active import mechanism for internalization.

Although the reported advantages of PROTACs over other therapeutics are remarkable, it is important to highlight their few universal drawbacks. A primary concern would be permeability: PROTACs are relatively larger than non-chimeric small molecules, so their transport across the plasma membrane can be challenging. The delivery of the compounds might turn out to be especially difficult in the case of crossing the trypanosomal cell and organellar membranes. Here, turning to smaller molecular weight compounds would be beneficial, and for example the CLIPTAC technology has been specifically developed to address this potential drawback. In this regard, molecular glues should also be especially advantageous owing to their small sizes and should be given due consideration when faced with issues related to the delivery of higher molecular weight degraders. A “Trojan horse” or packaging approach might also be developed in which TrypPROTACs are conjugated to or encapsulated by molecules improving their uptake or solubility (Liu et al., 2021; Jiang et al., 2023). It is however worth noting that degraders can be potent even in small doses, so that therapeutic efficiency might perhaps be achieved even at a low penetration to the site of action. As another concern, the design of degraders can also be laborious since it requires the synthesis of several analogs of ligands and linkers and the optimization of the linker that will bring about the formation of the desired ternary complex. The linker molecule is likely to be exposed to attack, which makes PROTACs and other degraders more unstable, thereby decreasing their half-lives (Troup et al., 2020). However, these challenges are worth tackling in the light of TPD advantages, such as the decreased emergence of drug resistance. Ultimately, no therapeutic approach is devoid of shortcomings, and targeted protein degradation may prove a turn-around in the fight against African trypanosomiasis. We hope that the guided discussion provided here will inspire researchers working on trypanocidal drug development to accelerate development of the first category of trypanocidal PROTACs.

## Figures and Tables

**Table 1:** Some Essential Proteins of African Trypanosomes and their Ligands.

S/N	TARGET	LIGANDS	REFERENCE
**1.**	Alternative oxidase & Glycerol kinase	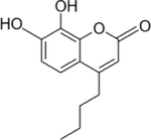	Balogun et al. (2019)
**2.**	Glyceraldehyde-3-phosphate dehydrogenase	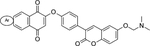	Belluti et al. (2014)
**3.**	Glycogen synthase kinase 3	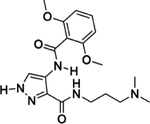	(Urich et al., 2014). Tear et al. (2020)
**4.**	Ornithine decarboxylase	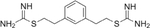	Smithson et al. (2010)
**5.**	Triosephosphate isomerase	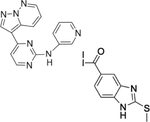	Vázquez-Raygoza et al. (2017)
**6.**	Trypanothione reductase	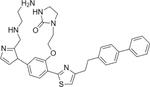	Revuelto et al. (2019)
**7.**	Rhodesain	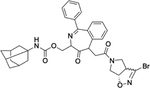	Ettari et al. (2016)
**8.**	UDP-GLC 4′ epimerase	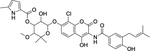	Durrant et al. (2010)

**Key:** *Ar* = *C4H4*.

## Data Availability

No data was used for the research described in the article.
